# Reversing abnormal hole localization in high-Al-content AlGaN quantum well to enhance deep ultraviolet emission by regulating the orbital state coupling

**DOI:** 10.1038/s41377-020-00342-3

**Published:** 2020-06-18

**Authors:** Li Chen, Wei Lin, Huiqiong Wang, Jinchai Li, Junyong Kang

**Affiliations:** 1 Engineering Research Center of Micro-nano Optoelectronic Materials and Devices, Ministry of Education; Fujian Provincial Key Laboratory of Semiconductor Materials and Applications; Collaborative Innovation Center for Optoelectronic Semiconductors and Efficient Devices; Department of Physics, Xiamen University, 361005 Xiamen, China; 2 Present Address: Ningbo Institute of Materials Technology and Engineering, Chinese Academy of Sciences, 315201 Ningbo, Zhejiang China

**Keywords:** Optical physics, Micro-optics

## Abstract

AlGaN has attracted considerable interest for ultraviolet (UV) applications. With the development of UV optoelectronic devices, abnormal carrier confinement behaviour has been observed for *c*-plane-oriented AlGaN quantum wells (QWs) with high Al content. Because of the dispersive crystal field split-off hole band (CH band) composed of *p*_*z*_ orbitals, the abnormal confinement becomes the limiting factor for efficient UV light emission. This observation differs from the widely accepted concept that confinement of carriers at the lowest quantum level is more pronounced than that at higher quantum levels, which has been an established conclusion for conventional continuous potential wells. In particular, orientational *p*_*z*_ orbitals are sensitive to the confinement direction in line with the conducting direction, which affects the orbital intercoupling. In this work, models of Al_0.75_Ga_0.25_N/AlN QWs constructed with variable lattice orientations were used to investigate the orbital intercoupling among atoms between the well and barrier regions. Orbital engineering of QWs was implemented by changing the orbital state confinement, with the well plane inclined from 0° to 90° at a step of 30° (referred to the *c* plane). The barrier potential and transition rate at the band edge were enhanced through this orbital engineering. The concept of orbital engineering was also demonstrated through the construction of inclined QW planes on semi- and nonpolar planes implemented in microrods with pyramid-shaped tops. The higher emission intensity from the QWs on the nonpolar plane compared with those on the polar plane was confirmed via localized cathodoluminescence (CL) maps.

## Introduction

AlGaN can be used in light-emitting diodes that can emit light ranging from ultraviolet (UV)-A to UV-C; in addition, it has excellent thermal conductivity, high physical hardness, high chemical resistance, and a high melting point. In the design of modern UV optoelectronic devices, confinement of carriers at the mesoscale is essential for efficient light emission. Conventional confinement exploits the heterostructure band offset between the Al_x_Ga_1−x_N well and the Al_y_Ga_1−y_N barrier (y > x > 0.5), wherein the effect of the atomic orbital orientation on the quantum confinement is ignored, even when the quantum structure is at the atomic scale. Therefore, the lattice discontinuity should be taken into account, especially for highly polarized lattice structures, such as AlGaN. Our previous investigation showed that the hole quantum confinement was abnormal in *c*-plane Al_0.60_Ga_0.40_N/Al_0.65_Ga_0.35_N QWs as an example of AlGaN QWs with an AlN mole fraction greater than 0.5 in one material^1^. This finding is contrary to the common belief that the carriers at the lowest quantum level are more tightly confined than those at higher quantum levels when the orbital configuration of the quantum level is not considered. In principle, the orbital configuration of the quantum level directly determines the probability of an optical transition between the band edge states. Since the valence band maximum (VBM) in an Al_x_Ga_1−x_N well with x > 0.5 is dominated by *p*_*z*_ orbitals and the heavy-hole (HH) and light-hole (LH) bands are composed of *p*_*x*_ and *p*_*y*_ states, the abnormal radiative interband transition in a QW is associated with the transition to the *p*_*z*_ state. Therefore, a comparable energy variation with its sign depending on the orbital configuration together with the confinement direction should be considered in addition to the band offset. It would be of interest if the underlying mechanism of orbital intercoupling based on the quantum confinement direction in a QW could be determined because this would provide a reliable starting point for orbital engineering that could be useful in the fabrication of other novel materials and optoelectronic devices.

In this work, the confinement dependence of the hole band on the constituent orbitals was calculated based on first principles and further investigated via experiments. To provide further context for understanding the orbital coupling in the QW, AlGaN QW models with high Al content were constructed with an AlN barrier and an Al_0.75_Ga_0.25_N well, and the coupling potential in the QW was calculated using the layer-decomposed density of states (DOS). Considering the quantum confinement enhancement, orbital engineering by varying the coupling orientation was proposed, and the resulting transition rate was evaluated. Orbital tuning was experimentally implemented by constructing QWs on semi- and nonpolar planes of microrods fabricated on patterned sapphire substrates (PSSs) using metalorganic vapor phase epitaxy (MOVPE). Cathodoluminescence (CL) mapping of the band edge emission was performed to further investigate orbital engineering with optimized confinement.

## Results and discussion

**Fig. 1:**
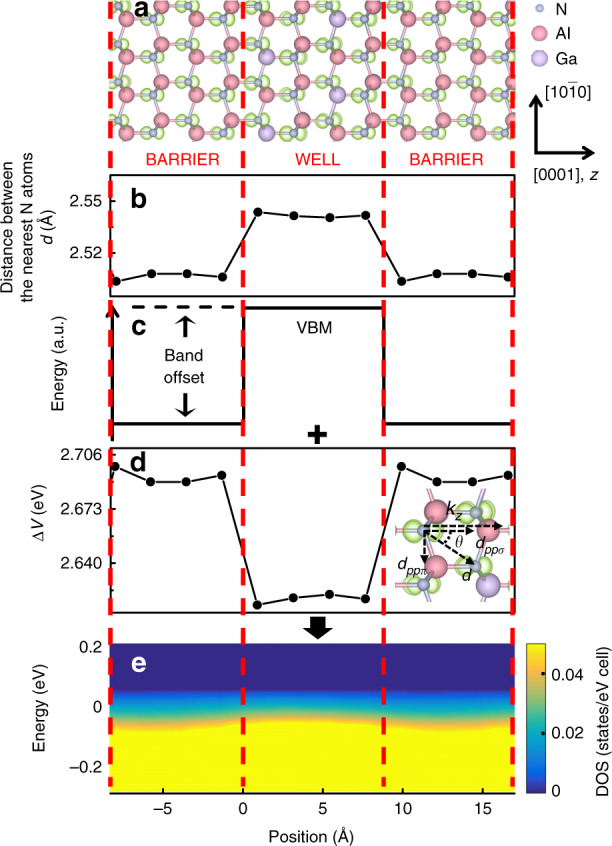
Electronic structures at the VBM for the QW contributed by the *p*_*z*_ orbital (*z* is perpendicular to the plane of the QW). **a** Surfaces (light green) corresponding to a charge density of 0.02 *e*/Å^3^ for the *p*_*z*_ orbital. The *z* axis is along the [0001] direction of the wurtzite lattice. **b** Distance *d* variation between the nearest N atoms. **c** Schematic of the band offset. The shape of the DOS drawn in **c** is illustrative and does not represent the actual band edge profile. **d** Coupling potential between *p*_*z*_ orbitals. The *p*_*z*_ orbital intercouplings can be decomposed into *pp*σ- and *pp*π-oriented components. **e** Layer- and *p*_*z*_ orbital-projected DOS at the VBM along the [0001] direction

**Fig. 2:**
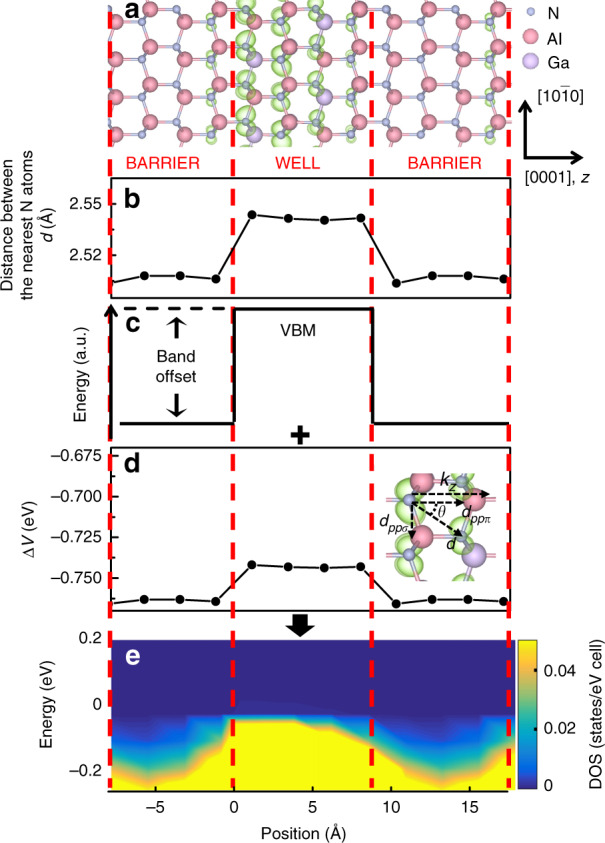
Electronic structures at the VBM for the QW contributed by the *p*_*x*_ orbital (*x* is parallel to the plane of the QW). **a** Surfaces (light green) corresponding to a charge density of 0.02 *e*/Å^3^ for the *p*_*x*_ orbital. The *z* axis is along the [0001] direction of the wurtzite lattice. **b** Distance *d* variation between the nearest N atoms. **c** Schematic of the band offset. The shape of the DOS drawn in **c** is illustrative and does not represent the actual band edge profile. **d** Coupling potential between *p*_*x*_ orbitals. The *p*_*x*_ orbital intercouplings can be decomposed into *pp*σ- and *pp*π-oriented components. **e** Layer- and *p*_*x*_ orbital-projected DOS at the VBM along the [0001] direction

The quantum confinement involved in the process of light emission is inherently linked to the VBM at the Γ point (*k* = 0) in the Brillouin zone for AlGaN. To examine the orbital orientation dependence of the quantum confinement at the VBM for Al_0.75_Ga_0.25_N/AlN QWs, isosurface plots of the orbital-projected partial charge density at the Γ-point for the *p*_*z*_ and *p*_*x*_ orbitals were obtained; these plots are illustrated in Figs. 1a and 2a, respectively. Figures 1b and 2b display the variation in the distance *d* between the nearest N atoms across the barriers and the wells. In addition, Figs. 1c and 2c schematically show the conventional well-barrier model derived based on band-offset theory. For *p*_*z*_ orbitals (*z* is perpendicular to the plane of the QW), there is a two-lobed-shaped charge distribution parallel to the *z* direction extending from the well to barrier planes, while for *p*_*x*_ orbitals (*x* is parallel to the plane of the QW), the corresponding charge distributions are localized in the well plane itself.

The constituent orbitals of the VBM, the $\left| {p_z} \right\rangle$ states, are commonly related to one of the spherical harmonics Y(*p*_*z*_) ∝ cos*θ*, where *θ* is the polar coordinate measured from the orientation of the $\left| {p_z} \right\rangle$ state. Y(*p*_*x*_) and Y(*p*_*y*_) have shapes identical to that of Y(*p*_*z*_) but are oriented with their maximum extensions along the *x*- and *y*-axes, respectively. It is well established that coupling between two *p*-states can be either head-on coupling, yielding *ppσ* coupling, or sideways coupling of parallel *p* orbitals on adjacent atoms, leading to *ppπ* coupling. The corresponding coupling energies, V_*ppσ*_ and V_*ppπ*_, between the orbitals can be given in a simplified manner by ${\mathrm{V}}_{pp\sigma } = {\mathrm{2}}.{\mathrm{22}}\frac{{\hbar ^2}}{{md^2}}$ and ${\mathrm{V}}_{pp\pi } = - 0.63\frac{{\hbar ^2}}{{md^2}}$, respectively, where *d* is the distance between the nearest atoms with $\left| {p_i} \right\rangle$ (*i* = *x*, *y*, or *z*) states^2^. Any *p*_*z*_ state having a mix of *ppσ* and *ppπ* couplings can be decomposed into *ppσ*- and *ppπ*-coupled components, both of which contribute to the energy variation ΔV in the band offset as follows:

1 $$\begin{array}{l} {\Delta}{\mathrm{V}}={\mathrm{V}}_{pp{\sigma}}{\cos}(\theta_{{\mathop{k}\limits^{\rightharpoonup}} {\cdot}{\mathop{d}\limits^{\rightharpoonup}}_{pp{\sigma}}})+{\mathrm{V}}_{pp{\pi}}{\cos}(\theta_{{\mathop{k}\limits^{\rightharpoonup}} {\cdot}{\mathop{d}\limits^{\rightharpoonup}}_{pp{\pi}}})\\={2.22}\frac{{\hbar}^{2}}{md^{2}_{pp{\sigma}}}{\mathrm{cos}}(\theta_{{\mathop{k}\limits^{\rightharpoonup}}_{z}{\cdot}{\mathop{d}\limits^{\rightharpoonup}}_{pp{\sigma}}}) -0.63\frac{{\hbar}^2}{md^2_{pp{\pi} }}\cos(\theta_{{\mathop{k}\limits^{\rightharpoonup}}_{z} {\cdot}{\mathop{d}\limits^{\rightharpoonup}}_{pp{\pi}}}) \end{array}$$

where *k*_*z*_ is the hole momentum in the conducting direction, which is in line with the confinement direction normal to the well plane.

It can be observed in the inset of Fig. 1d that although the *p*_*z*_ orbitals of the CH band are not oriented exactly in line, the orbital intercouplings that are not purely *ppσ* or *ppπ* coupling directed in the *d* direction can be decomposed into two components as in Eq. (1). The $\left|{p_z}\right\rangle$ state of the CH band on one atom is end-to-end coupled with the $\left|p_{z}\right\rangle$ state on another atom along *k*_*z*_ out of the well plane, with ${\mathrm{V}}_{pp\sigma }\cos (\theta_{{\mathop{k}\limits^{\rightharpoonup}} \cdot {\mathop{d}\limits^{\rightharpoonup}}_{pp{\sigma}}}) = 2.22\frac{{\hbar^2}}{{md^2_{pp\sigma }}}\cos (\theta_{{\mathop{k}\limits^{\rightharpoonup}}_{z} \cdot {\mathop{d}\limits^{\rightharpoonup}}_{pp{\sigma}}})$, while V_*ppπ*_ coupling is formed between the parallel $\left| {p_z} \right\rangle$ states in the well plane. In addition, the component of ${\mathrm{V}}_{pp\pi }{\mathrm{cos}}\left( {\theta _{\mathop{k_z}\limits^{\rightharpoonup} \cdot \mathop{d_{pp\pi }}\limits^{\rightharpoonup} }} \right)$ along *k*_*z*_ is 0.

Based on lattice parameters calculated using first principles simulations, the energy variation between *p*_*z*_ states derived from Eq. (1) is shown in Fig. 1d. Combining the conventional barrier-well model shown in Fig. 1c with the coupling effect shown in Fig. 1d, the overall energy variation with the *p*_*z*_ state is schematically depicted in Fig. 1e, which serves as a useful guide for understanding the electronic structure of the QW. From Fig. 1b, d, it can be observed that the different material composition in the well leads to a larger atomic separation *d* and a significant decrease in the *p*_*z*_ energy compared to those in the barrier. The increase in the energy variation in the barrier compensates for the band offset in the QW. Accordingly, the position-dependent projected DOS of *p*_*z*_ orbitals in Fig. 1e, which reflects the overall potential in the QW, has a nearly flat energy distribution along the QW structure at the lowest valence energy level, leading to a very small potential barrier in the quantum structure. This observation is consistent with our previous report^1^ regarding the abnormal radiative emissions from Al_0.75_Ga_0.25_N QWs oriented on the (0001) plane and occurs because of the dispersive CH band caused by *p*_*z*_ orbitals delocalized into the barrier, which provides little quantum confinement in the QW.

Similar analyses were conducted for the *p*_*x*_ orbital-projected DOS, as shown in Fig. 2d, e. In contrast to the case of the *p*_*z*_ orbital, V_*ppπ*_ coupling between parallel $\left| {p_x} \right\rangle$ or $\left| {p_y} \right\rangle$ states in the HH/LH bands becomes the dominant contributor along ${\mathop{k}\limits^{\rightharpoonup}}_z$, with a negative term of $V_{pp\pi }\cos (\theta _{\mathop{k}\limits^{\rightharpoonup} \cdot {\mathop{d}\limits^{\rightharpoonup}}_{pp\pi }}) = - 0.63\frac{{\hbar ^2}}{{md_{pp\pi }^2}}\cos (\theta _{{\mathop{k}\limits^{\rightharpoonup}}_z \cdot {\mathop{d}\limits^{\rightharpoonup}}_{pp\pi }})$, as shown in the inset of Fig. 2d. Consequently, the barrier potential of the *p*_*x*_ and *p*_*y*_ states increases due to *ppπ* coupling. In particular, from Fig. 2e, it can be observed that the enhanced barrier provides adequate confinement for *p*_*x*_ and *p*_*y*_ states at the valence band edge, in agreement with the quantum level character in the band structure of the QW.

Based on the abovementioned results, band offset compensation is primarily contributed by the V_*ppσ*_ term $\left( { = {\mathrm{2}}.{\mathrm{22}}\frac{{\hbar ^2}}{{md_{pp\sigma }^2}}\cos (\theta _{\mathop{k}\limits^{\rightharpoonup} \cdot {\mathop{d}\limits^{\rightharpoonup}}_{pp\sigma }})} \right)$ for the well region, while barrier enhancement is dominated by the V_*ppπ*_ term $\left( { = - 0.63\frac{{\hbar ^2}}{{md_{pp\pi }^2}}\cos (\theta _{\mathop{k}\limits^{\rightharpoonup} \cdot {\mathop{d}\limits^{\rightharpoonup}}_{pp\pi }})} \right)$, aside from the contribution of the lattice distance *d*, which is usually small in high-Al-content AlGaN wells.

It is clear from Eq. (1) that although a variation in the distance *d* in the well can lead to a change in the energy variation, the sign of the value will not be affected. In addition, the term $\cos (\theta _{\mathop{k}\limits^{\rightharpoonup} \cdot {\mathop{d}\limits^{\rightharpoonup}}_{pp\sigma /\pi } })$ becomes zero when the conducting direction $\mathop{k}\limits^{\rightharpoonup}$ is orthogonal to ${\mathop{d}\limits^{\rightharpoonup}} _{pp\sigma /\pi }$, while it becomes nonzero when the conducting direction $\mathop{k}\limits^{\rightharpoonup}$ is tilted in the direction of ${\mathop{d}\limits^{\rightharpoonup}} _{pp\sigma /\pi }$. Owing to these coupling features, the confinement barrier is affected by competition between *ppσ* and *ppπ* couplings when the conducting direction $\mathop{k}\limits^{\rightharpoonup}$ is in line with the confinement direction. However, by varying the conducting direction $\mathop{k}\limits^{\rightharpoonup}$—which could lead to the values of $\cos (\theta _{\mathop{k}\limits^{\rightharpoonup} \cdot {\mathop{d}\limits^{\rightharpoonup}}_{pp\sigma /\pi } })$ varying between 0 and 1—the sign of the energy variation can be modified. In light of the energy variation dependence based on the confinement direction, the *ppπ* coupling is expected to play a dominant role in the orbital coupling (${\mathrm{V}}_{pp\pi }\cos (\theta _{\mathop{k}\limits^{\rightharpoonup} \cdot {\mathop{d}\limits^{\rightharpoonup}} _{pp\pi }})$ is larger than V_*ppσ*_$\cos (\theta _{\mathop{k}\limits^{\rightharpoonup} \cdot {\mathop{d}\limits^{\rightharpoonup}} _{pp\sigma }})$), and the resulting negative energy variation would then lead to a barrier enhancement beneficial for optoelectronic devices with sufficient quantum confinement.

**Fig. 3:**
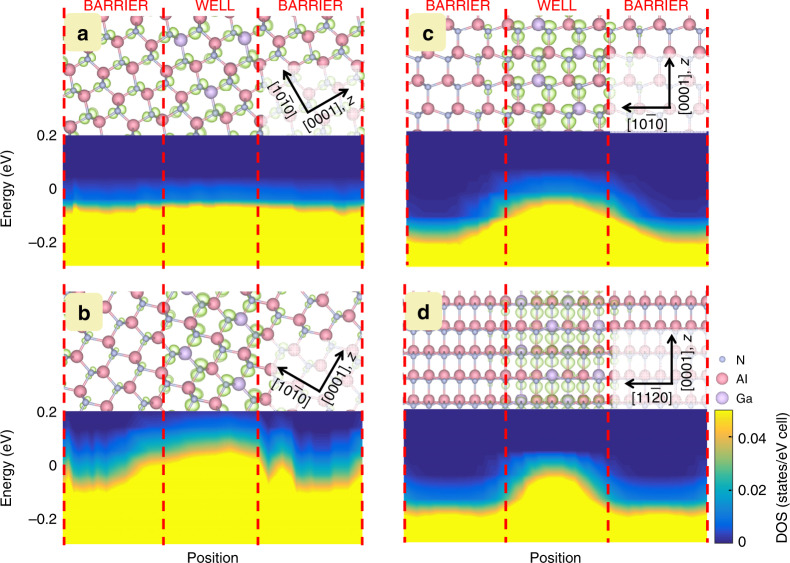
Electronic structures at the VBM for the AlGaN/AlN QWs with inclined well planes. Surfaces (light green) corresponding to a charge density of 0.02 *e*/Å^3^ for the *p*_*z*_ orbital of the AlGaN/AlN QW models constructed with different well orientations of **a** 30°, **b** 60°, and **c**, **d** 90° together with the layer- and *p*_*z*_ orbital-projected DOSs at the VBM along the [0001] direction. The 90° inclined QWs are constructed on the (10$\bar 1$0) plane and (11$\bar 2$0) plane in **c**, **d**, respectively

To further clarify the characteristics of the orbital-state coupling, the quantum confinement was modulated by varying the confinement direction by inclining the well plane away from the (0001) plane. Compared to the QW parallel to the (0001) plane, the delocalized *p*_*z*_ orbitals (Fig. 3a) experience volume shrinkage in the barrier region, and the corresponding layer-decomposed DOS of the *p*_*z*_ state shows a rather small potential barrier in the valence band. As the inclination angle of the well plane in the QW increases, the charge distribution of the CH band shows better confinement because of the presence of a distinct potential barrier, as shown in the layer-decomposed DOS of the *p*_*z*_ state (Fig. 3b). For the QW on the nonpolar (10$\bar 1$0) plane, as depicted in Fig. 3c, the *p*_*z*_ state is well confined in the QW, with an increased barrier potential in the well at the VBM. The enhanced quantum confinement for an inclination angle of 90° is further emphasized by the QW on the nonpolar (11$\bar 2$0) plane, as shown in Fig. 3d, which indicates that inclining the well plane towards the orbital orientation reduces *ppσ* coupling. Thus, *ppπ* coupling between the *p* orbitals in the well plane reinforces the potential barrier and enhances quantum confinement for the quantum level in nonpolar high-Al-content AlGaN QWs.

**Fig. 4:**
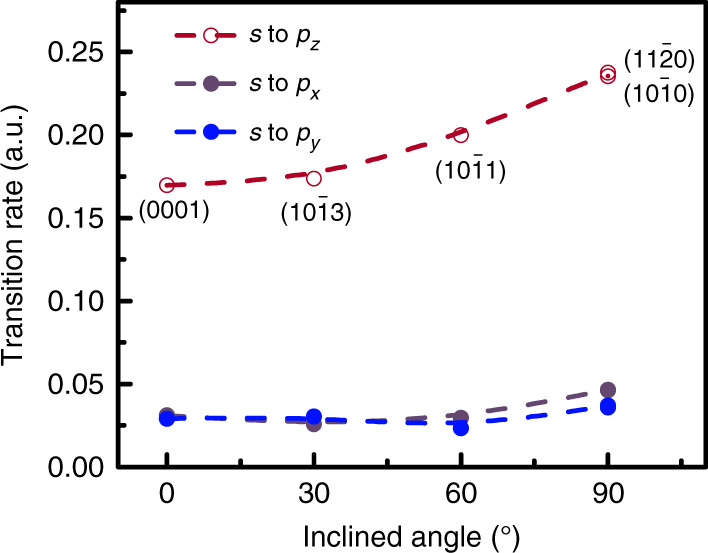
Transition rate of the *s* to *p* states for different inclination angles of the well plane. The purple, blue, and dark red lines represent transitions from the *s* to *p*_*x*_ state, *s* to *p*_*y*_ state, and *s* to *p*_*z*_ state, respectively

In general, the confinement in QWs determines the transition rates of electrons from the conduction band minimum (CBM) to the VBM; these transition rates can be calculated using Fermi’s golden rule, $\Gamma _{sp}(\omega _{ij}) = \left| {M_{ij}} \right|^2\rho (\omega _{ij})$, where *M*_*ij*_ is the transition matrix element and *ρ*(*ω*_*ij*_) is the joint DOS for an electron in initial state *i* recombining with a hole in state *j*. The calculated transition rates from the *s* state at the CBM to the *p*_*x*_, *p*_*y*_, and *p*_*z*_ states at the VBM in the QWs are shown in Fig. 4. With increasing inclination angle of the well plane, the transition rate dramatically increases, which demonstrates that confinement in QWs dominates the transition rates of electrons from the CBM to VBM as well as the emission intensity. The transition rates for the semipolar planes (10$\bar 1$3) and (10$\bar 1$1) are 1.02 and 1.17 times and those of the nonpolar planes (10$\bar 1$0) and (11$\bar 2$0) are 1.40 and 1.40 times that for the polar (0001) plane, respectively. It should be noted that the highest transition rates are observed for the QWs on the nonpolar planes. Although the transition rates of the *p*_*x*_ and *p*_*y*_ states at the VBM vary only slightly, they are still ~5 times smaller than that of the *p*_*z*_ state. Consequently, if there is any emission from the QW, then the emission intensity from the *p*_*z*_ state on non- and semipolar planes will dominate, instead of those from the *p*_x_ and *p*_y_ states. This also indicates that QWs constructed on the non- and semipolar planes would have higher internal quantum efficiency than those on the polar plane. This enhanced transition rate confirms the improved light emission because of orbital engineering.

**Fig. 5:**
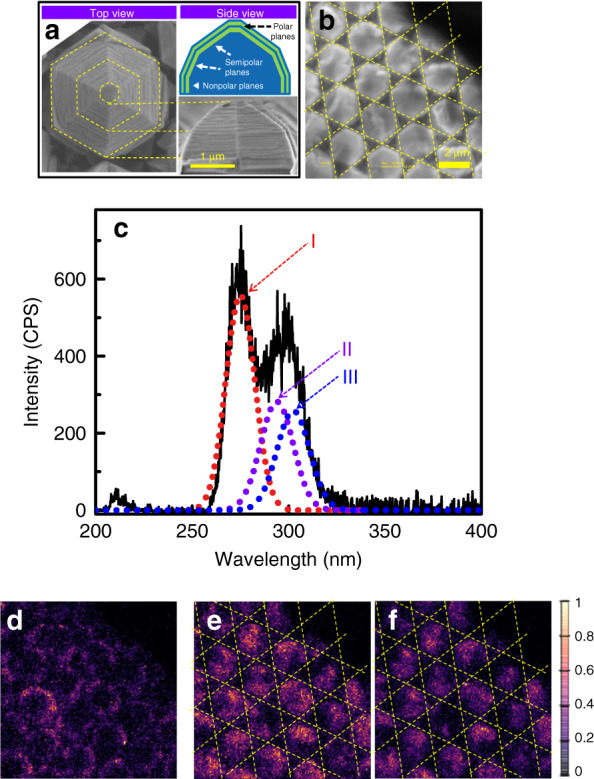
CL characterization of QWs with inclined well planes. **a** Top- and side-view SEM images of a typical microrod together with a schematic of the microrod structure. **b** Top-view SEM image of a region studied using CL. **c** CL spectrum of the microrod array. **d** CL map recorded for peak I from nonpolar planes. **e** CL map recorded for peak II from semipolar planes. **f** CL map recorded for peak III originating from polar planes

Furthermore, this concept of orbital engineering through inclination of the well plane was demonstrated experimentally by constructing QWs on semi- and nonpolar planes. QW planes with inclination angles of 30° and 60° correspond to the (10$\bar 1$3) and (10$\bar 1$1) planes, respectively, while the angle of 90° is associated with the (10$\bar 1$0) or (11$\bar 2$0) plane. QWs on semi- and nonpolar planes were implemented by selective growth on PSSs using MOVPE. Figure 5a shows the top view and a side view of one typical hexagonal microrod, and the morphology of the microrod array is shown in Fig. 5b. It can be seen that a microrod contains a limited area of the polar plane on top, smaller than those of the inclined and sidewall facets formed by semi- and nonpolar planes. The larger areas of the semi- and nonpolar planes suggests that QW formation on the (0001) plane was suppressed, resulting in the main growth on semi- and nonpolar planes, which is favourable for CH band confinement.

The optical properties of the microrods were evaluated via CL measurements at room temperature. The detected CL spectrum in Fig. 5c consists of emission peaks centred at 277, 295, and 300 nm, which are denoted I, II, and III, respectively. Considering the inclination-angle-dependent transition rates depicted in Fig. 4, the emission from the *p*_*z*_ state on non- and semipolar planes should be dominant in the CL spectrum, rather than those from the *p*_*x*_ and *p*_*y*_ states. The broadened line shape for overlapping peaks II and III suggests that the luminescence from QWs on different semipolar planes at the top of the microrods contributes to the observed emissions. To confirm this observation, CL mappings were conducted at wavelengths of 277, 295, and 300 nm (i.e., at peaks I, II, and III). These mappings are shown in Fig. 5d–f, respectively. The strong emission that is represented by peak I primarily arises from the sidewall facets of the microrods, as shown in Fig. 5d. In comparison, emission peak II is from the semipolar planes, and emission peak III is predominantly from the polar planes, as shown in Fig. 5e, f, respectively. These observations validate the theoretical predictions that the confinement barrier is dependent not only on the band offset but also on the orbital couplings.

The aforementioned *ppπ* coupling contributes a negative term to the energy variation for the QWs on the nonpolar planes, which directly lowers the CH quantum level and increases the band gap in the well. Furthermore, the energy variation in the well is less negative than that in the barrier, which leads to enhancement of the confinement barrier for the CH band and indirectly reduces the CH quantum level. Therefore, the corresponding wavelengths are shorter than those of emissions from the QWs on the semipolar and polar planes of the same well width. The shorter wavelength for the nonpolar planes indicated by the CL maps agrees with the *ppπ* coupling features. Because the CL spectrum is captured from the pyramid top, the emission intensities from the nonpolar planes on the sidewall would be weaker than those from the semipolar planes on the top. However, the observation of stronger emission intensities from the sidewall provides evidence of higher transition rates in the QWs on the nonpolar planes. All the aforementioned emission behaviours demonstrate that aligning the QW plane on a nonpolar plane is beneficial in reducing the *ppσ* coupling and increasing light emission related to the CH band.

The finite “potential well” defined by the traditional band offset concept is inadequate for orbital engineering based on quantum confinement. The pivotal role of orbital intercoupling is important to consider because of the orientation sensitivity of active valence *p* states to the confinement direction in the quantum structure, which distorts the symmetric rectangular potential of the well, especially in the case of materials with strong polarization. In general, awareness of the orbital intercoupling and its influence on the well potential provides a new perspective on the construction of heterostructures and superlattices, which could enable us to take advantage of multiple useful properties of materials via polarization of orbitals. For example, because of the *p*_*z*_ character in the highest valence band of wurtzite MgO^3^, research related to ZnMgO-based quantum structures might also find control of the valence orbital configuration useful. High-temperature superconductivity is observed in charge-transfer compounds characterized by strong hybridization between oxygen 2*p* and transition metal 3*d* states and with complex electronic configurations^4,5^. Quite recently, magic-angle graphene superlattices have attracted significant interest owing to their unconventional superconductivity^6,7^. This emerging superconductivity hints at the presence of interlayer orbital coupling between stacking single layers of graphene composed of two-dimensional van der Waals materials. Thus, it could be expected that, beyond the optical properties discussed in this work, orbital engineering with confinement and strain could also lead to enhanced superconductivities and other desirable functional properties in heterostructures and superlattices and even to two-dimensional honeycomb structures derived from Group IV elements and Group III–V and II–VI compounds.

In conclusion, this work demonstrates the crucial role of atomic orbitals in quantum confinement. The energy variation induced by the orbital intercoupling among atoms between the well and barrier regions can be comparable to the band offset obtained from the bulk properties of the well and barrier materials. Our first principles calculations for high-Al-content AlGaN show that the band offset compensation is primarily contributed by the *ppσ* term, while the barrier enhancement is dominated by the *ppπ* term. The energy variation dependence on the orbital coupling orientation with respect to the quantum confinement provides an avenue for research on innovative quantum confinement design by varying the confinement direction. The interaction between the charge confinement of the hole band and orbital coupling modulation is illustrated by inclining the well plane by constructing wells on semi- and nonpolar planes. In particular, by inclining the well plane, the distributions of the *p* states at the VBM further confirmed the enhanced quantum confinement in the well with a higher well potential. The spontaneous emission related to the hole orbitals at the VBM improved as the charge was confined in the QW. The QWs constructed on the semi- and nonpolar planes showed higher internal quantum efficiencies than those constructed on the polar plane. Experimental characterization of the optical properties of the semi- and nonpolar planes was conducted using hexagonal microrods grown on PSSs via MOVPE. Based on the observed microrod morphologies, the emission peaks most likely originated from band edge emission from planes with different orientations rather than from multiple emissions from the polar plane. The CL intensities at shorter wavelengths were generally higher than those at longer wavelengths, even though CL characterization was performed from the pyramid top, which provides evidence for the higher emission intensities from QWs on nonpolar planes. These results provide useful guidelines for the design of high-performance devices based on high-Al-content AlGaN. In addition, the concept of orbital engineering could also be implemented in other functional devices.

## Materials and methods

Theoretically, first-principles simulations were performed using the Vienna ab initio Simulations Package (VASP)^8,9^, which implements density functional theory with the local density approximation. The projector augmented wave approach for the Perdew-Wang 91 generalized gradient approximation^10,11^ was used to describe the interaction between ions and electrons. Ga 3*d* electrons were included in the valence band. A plane wave cutoff energy of 500 eV and an 8 × 8 × 8 Monkhorst-Pack *k*-point mesh of the Brillouin zone were used for all the calculations^12^. The QW models comprised an AlN barrier and an Al_0.75_Ga_0.25_N well, each with a thickness of ~1 nm. Geometric optimization was performed by relaxing all degrees of freedom using the conjugate gradient algorithm, in which the total energy converged within 1 meV. The *sp*- and site-projected wave function characteristics of each band were evaluated, and the local partial DOS was calculated. The optimized structure obtained from the VASP simulation was then used as input for additional calculations of the coupling between orbitals. The CBM and VBM contributing to quantum confinement were composed of *s* and *p* orbitals, respectively. Because *s* orbitals are spherical, the present study focused on the role of directional *p* orbitals, which are sensitive to the orbital configuration in the QW. To identify the constituent states at the VBM, the DOS was projected onto the *p* orbitals of atoms in the local coordinates (*xyz*) with the *z* axis parallel to the *c* axis of the crystal and the *x* axis parallel to the [10$\bar 1$0] crystalline direction. The layer-decomposed DOSs calculated for various configurations of the QWs are shown in Figs. 1e, 2e and 3, with the DOS value represented by a continuous colour variation from blue to yellow. The lateral axis is the position, with the origin located at the lower interface of the well, and the axis direction is chosen along the normal direction of the QW. The partial charge density was calculated for the bands, which was closely related to the associated optical transitions.

Experimentally, epitaxial growth was conducted on *c*-plane PSSs via MOVPE (Thomas Swan 3 × 2″ CCS). During MOVPE growth, trimethylgallium (TMGa), trimethylaluminium (TMAl), and ammonia (NH_3_) were used as gallium, aluminium, and nitrogen precursors, respectively. After pre-deposition heat treatment under H_2_, nitridation of the sapphire substrate was performed at 570 °C for 200 s. Subsequently, a 10-nm-thick AlN buffer was deposited at a temperature of approximately 875 °C prior to the growth of a 4-μm AlN template at a temperature of 1210 °C with the desired crystal quality based on the hierarchical growth method, in which selected growth of monomers was induced by modulating the chemical potential with the aid of varying growth conditions in N-rich and Al-rich environments^13^. Five periods of 3-nm Al_0.45_Ga_0.55_N/10-nm Al_0.55_Ga_0.45_N QWs targeted for high Al content on average were grown at 1210 °C under a pressure of 75 Torr with nitrogen as a carrier gas using hierarchical growth units processed to achieve structures with high crystalline quality and interfacial abruptness^14,15^. The optical and structural properties were assessed using CL and scanning electron microscopy.
